# oxLDL-mediated cellular senescence is associated with increased NADPH oxidase p47phox recruitment to caveolae

**DOI:** 10.1042/BSR20180283

**Published:** 2018-06-12

**Authors:** Jing Wang, Yuzhi Bai, Xia Zhao, Jing Ru, Ning Kang, Tian Tian, Liying Tang, Yun An, Pei Li

**Affiliations:** 1 Department of Geriatrics, Beijing Chaoyang Hospital Affiliated to Capital Medical University, Beijing 100043, China; 2 Institute for Immunology, Department of Basic Medical Sciences, School of Medicine, Tsinghua-Peking Joint Center for Life Sciences, Tsinghua University, Beijing 100084, China

**Keywords:** atherosclerosis, cell senescence, caveolin-1, p47phox

## Abstract

Atherosclerosis develops as a consequence of inflammation and cell senescence. In critical factors involved in the atherosclerotic changes, reactive oxygen species (ROS) generation is considered a leading cause. While NADPH oxidases, particularly NOX2, are the main sources of ROS, how they are regulated in the disease is incompletely understood. In addition, how caveolae, the membrane structure implicated in oxLDL deposition under vascular endothelia, is involved in the oxLDL-mediated ROS production remains mostly elusive. We report here that macrophages exposed to oxLDL up-regulate its caveolin-1 expression, and the latter in turn up-regulates NOX2 p47phox level. This combination effect results in increased cellular senescence. Interestingly, oxLDL treatment causes the p47phox residing in the cytosol to translocate to the caveolae. Immunoprecipitation assays confirms that cavelin-1 is in high degree association with p47phox. These results suggest caveolin-1 may serve as the membrane target for p47phox and as a switch for ROS production following oxLDL exposure. Our results reveal a previously unknown molecular event in oxLDL-mediated cellular ageing, and may provide a target for clinical intervention for atherosclerosis.

## Introduction

Atherosclerosis is a disease of ageing and inflammation. It is a pathological consequence of vascular plaque formation. At late stages, a fraction of plaques rupture, leading to thrombosis, the clinical manifestation of cardiovascular pathologies. The plaque formation is complex, driven by local lesion, inflammatory milieu, cholesterol deposition, and other factors [1]. In this process, reactive oxygen species (ROS) are involved in at multiple stages of pathogenesis [2,3].

Amongst many sources of ROS, NADPH oxidase is relatively well-characterized [4]. The dominant vascular NADPH oxidase isoform is NOX2 [5]. NOX2 has several subunits. Gp91 (CYBB, mediating the electron transfer in the oxidase) and gp22 (CYBA) are membrane-bound and collectively form cytochrome *b558*. The regulatory subunits p47phox, p67phox, p40phox, and a GTPase remain in the cytosol until activation. Activation signals can turn on NOX2, via extensive phosphorylation of p47phox. In the resting cells, p47phox is in a state of auto-inhibition. Its tandem SH3 domains bind to its own C-terminal polybasic region (PBR) sequence [5]. A diverse collection of stimulations lead to p47 phosphorylation at several residues, leading to its structural opening. The sites of phosphorylation often reflect the nature of distinct stimulation and likely to fine-tune the consequences. Following the phosphorylation, the cytosolic subunits then move to the membrane to form the functional cytochrome *b558*.

Regarding the membrane structures that are involved in atherosclerosis, one of the most studied is caveolae [6–8]. They are 50–100 nm membrane structures that have been regarded as one of the subset of lipid rafts due to their morphology and lipid composition [9]. Upon stimulation, they may form flask-like invagination into the cell and become internalized. Distinct from conventional endophagosomes, there is little evidence that caveolae internalization is associated with endosome/lysosome formation. Rather they appear to be a transport system that deliver its cargos to the endocytic system [10]. With regard to atherosclerosis, the caveolae system has been implicated in transporting LDL to cross the epithelial cells and in the deposition of cholesterol in vascular walls [11–13]. However, mounting evidence suggests that caveolae is involved in signal transduction. The structural formation of caveolae is dependent on caveolin-1 expression which forms the scaffold to support the flask-like membrane indentation. However, an interesting property of caveolin-1 is its ability to interact with a large number of proteins and lipid species [14]. Caveolin-1 has an internal sequence that is buried in the inner leaflet with both N- and C-termini free in the cytosol. A caveolin scaffolding domain (CSD) is involved in multiple protein bindings, such as src family kinases, MAPKs and NO synthases, and those bindings can result in both activation and inhibition [15,16].

As both NOX2 and caveolin-1 are involved in senescence, it is an interesting study how p47phox and caveolin-1 interact with each other in the ageing process, particularly in atherosclerosis. In this brief report, we demonstrate that two events are at the center of oxLDL-mediated ageing. We first show that oxLDL mediates caveolin-1 and p47phox expression in a chain event whereby the absence of caveolin-1 leads to diminished p47phox protein expression. The caveolin-1 expression is associated with increased oxidative response and cellular senescence. Depletion of caveolin-1 blocks these events. Second, we show that upon oxLDL stimulation, caveolin-1 is in physical association with p47phox and ROS production appears to be confined in caveolae. These findings reveal new details how oxLDL may contribute to atherosclerotic pathology and may suggest new targets of clinical intervention.

## Materials and methods

### Cell culture

RAW264.7 cells were cultured in RPMI-1640 medium (HyClone, America) supplemented with 10% FBS (Gibco, America), 100 U/ml penicillin and 100 mg/ml streptomycin, 10 mM HEPES and 50 μM β-mercaptoethanol. HEK293T cells were maintained in DMEM supplemented with 10% FBS and the antibiotics described above. For the experiment, cells were growth arrested in serum-free medium for 12 h. To verify the changes of caveolin-1 in senescent cells, RAW264.7 were incubated with different concentrations of oxLDL (0–80 μg/ml) for 24 h or with different times. To explore the underlying mechanisms of caveolin-1 in senescent cells, ctrl shRNA or cav1 shRNA transfected Raw264.7 cells were treated with oxLDL or left untreated.

### Knockdown of gene expression using shRNA

Five shRNA clone targetting Cav1 were purchased from Sigma: F11 CCGGCCAGTTAGATTTAGGGATTTACTCGAGTAAATCCCTAAATCTAACTGGTTTTTG; F12CCGGCCGCTTGTTGTCTACGATCTTCTCGAGAAGATCGTAGACAACAAGCGGTTTTTG; G1 CCGGCGACGTGGTCAAGATTGACTTCTCGAGAAGTCAATCTTGACCACGTCGTTTTTG; G2 CCGGTGAAGCTATTGGCAAGATATTCTCGAGAATATCTTGCCAATAGCTTCATTTTTG; G3 CCGGGCTTCCTGATTGAGATTCAGTCTCGAGACTGAATCTCAATCAGGAAGCTTTTTG.

Lentiviral particles were packaged in 293FT using Lipofectamine 2000 reagent (Invitrogen, America). shRNA plasmid was transfected with the packing plasmid pCMVΔ8.9 and the envelope plasmid pHCMV-G. Scrambled shRNA was used as a control. Forty-eight hours later, supernatant containing viral particles was collected with 2000 rpm centrifugation for 5 min at 4°C. Raw264.7 cells were seeded at 5 × 10^5^ per well in six-well plates (approximately 70% confluent). After 12 h, 0.5 ml culture supernatant containing the lentivirus was added to the wells in the presence of 8 μg/ml Polybrene. The plates then were centrifuged at 2500 rpm at 32°C for 1 h and returned to the culture. Twenty-four hours later, fresh medium containing 3 μg/ml puromycin was added to the plates for selection and the puromycin medium was changed every other day. Stable shRNA expressing cell line was selected by 7 days. The *caveolin-1* mRNA level in different shRNA transfected groups was examined by RT-PCR.

### RNA analysis

Total RNA was isolated from RAW264.7 using Takara TRIzol reagent. Reverse transcription was performed with Takara PrimeScript RT kit with gDNA Eraser. It was followed by RT-PCR analysis. Amplification primers for caveolin-1 are as follows: F CTACAAGCCCAACAACAAGGC and R AGGAAGCTCTTGATGCACGGT. RT-PCR analysis for β-actin was carried out using Forward CTGGACTTCGAGCAAGAGATG and Reverse TGATGGAGTTGAAGGTAGTTTCG primer pairs. The RT-PCR was performed on Bio-Rad C1000 Touch™ Thermal Cycler using SYBR green mix (Genestar) according to manufacturer’s instructions.

### Western blotting

For caveolin-1 and p47phox protein detection, the cells were lysed in the lysis buffer (50 mM Tris/HCl, pH 7.4, 0.1 mM EDTA, and protease-inhibitor mixture) and the membrane fraction of cell lysate was prepared by ultracentrifugation (100000×***g*** for 1 h at 4°C). Protein concentration was quantitated with BCA protein quantity assay kit (Applygen, Beijing, China). Equal amounts of proteins were loaded on to SDS/polyacrylamide gel (10 or 12% gel), separated by electrophoresis and transferred on to PVDF membranes. After blocking with 5% skim milk in TBS with 0.05% Tween 20 (TBS-T) for 1 h at room temperature, membranes were incubated with mouse anti-p47phox (1:1000), rabbit anti-caveolin-1 (1:1000), rabbit anti-actin (1:1500), mouse anti-GAPDH (1:1500), overnight at 4°C. All primary antibodies were from Santa Cruz Biotchnology except for the last one from BioLegend. Membranes were washed three times in TBS-T for 10 min and incubated with HRP-labeled second antibodies (1:8000) for 1 h at room temperature, the bands of interest were detected using an enhanced chemiluminescent stain.

### Senescence assay

A commercial kit for β-galactosidase (β-gal) staining was used to assess the RAW264.7 senescence induced by ox-LDL (Beyotime Biotechnology, China). For cell senescence assay, ctrl shRNA or caveolin-1 shRNA transfected Raw264.7 cells were treated with oxLDL or left untreated. Twenty-four hours later, cells were washed three times with PBS when grown up to 80% confluence. The fixation solution which included 2% formaldehyde, 0.2% glutaraldehyde was added to cells at room temperature. After 5 min, cells were washed three times with PBS. Then, freshly prepared 1 ml SA-β-gal was used to stain the cells at 37°C for 12–16 h. The blue precipitates can be seen in the cytoplasm. For each sample, six fields were randomly selected. The percentage of positive cells was calculated.

### ROS measurement

The level of intracellular ROS generation was determined by measuring the oxidative conversion of DCFH-DA (Sigma) to fluorescent dichlorofluorescin (DCF). Briefly, RAW264.7 (5 × 10^5^ cells) were treated with 60 μg/ml oxLDL for 6 h while ROS inducer was used as the positive control. Cells were digested by 0.25% trypsin (Invitrogen) with 0.01% EDTA and terminated by medium containing 10% FBS. Cells were washed three times with PBS, and fluorescent intensity was measured by FBS. Mean fluorescence intensity (MFI) was used to indicate the ROS level.

### Gelatin zymography

For gelatin zymography assay, cell supernatants were quantitated by Bio-Rad reagent for protein concentration and 30 μg protein was loaded into SDS/PAGE (10% gel) supplemented with 1% gelatin. After electrophoresis, gels were washed twice for 30 min at room temperature in 2.5% Triton X-100 to remove the SDS. Then, gels were stained with 0.05% Coomassie Brilliant Blue R250 in 30% methanol and 10% acetic acid at 37°C for 3 h, then the gels were incubated with destaining buffer until bands can clearly be seen. Finally, gels were scanned by digital gel imaging system.

### Co-immunoprecipitation

For immunoprecipitation assay, ctrl shRNA or caveolin-1 shRNA transfected RAW264.7 cells were treated with oxLDL or left untreated. Four hours later, cells were lysed with NP-40 lysis buffer (50 mM HEPES pH 7.4, 150 mM NaCl, 1% NP-40, 1 mM EDTA, 1 mM PMSF, 1 mM NaF, 1 mM NaVO_3_, and protease inhibitor cocktails). The cell lysates were incubated with antibody–bead mixtures overnight at 4°C. Beads were then collected by low speed centrifugation (10000×***g***, 5 min) and washed four times with immunoprecipitation lysis buffer (300 mM NaCl added). Pellets were then subjected to the Western blotting analyses.

### Fluorescent imaging

For immune fluorescent staining, cells were washed three times with cold PBS and fixed with 4% paraformaldehyde for 20 min at room temperature. Then, cells were washed three times with PBS and permeabilized with 0.2% Triton X-100 in PBS for 30 min. After permeabilization, cells were washed three times with PBS and blocked by adding house serum during 30 min at room temperature. Then samples were stained with anti-p47phox and anti-caveolin-1 antibodies, washed with PBS for three times, incubated with the second antibodies and washed subsequently. Finally, samples were mounted and observed under a confocal laser-scanning microscope.

### Statistics

All results were presented as means ± S.D. Statistical differences amongst groups were assessed using ANOVA. Post hoc comparisons were performed with Tukey’s test to indicate the significant variations between groups. A value of *P*<0.05 was considered to be statistically significant.

## Results

### oxLDL promotes caveolin-1 production

**Figure 1:**
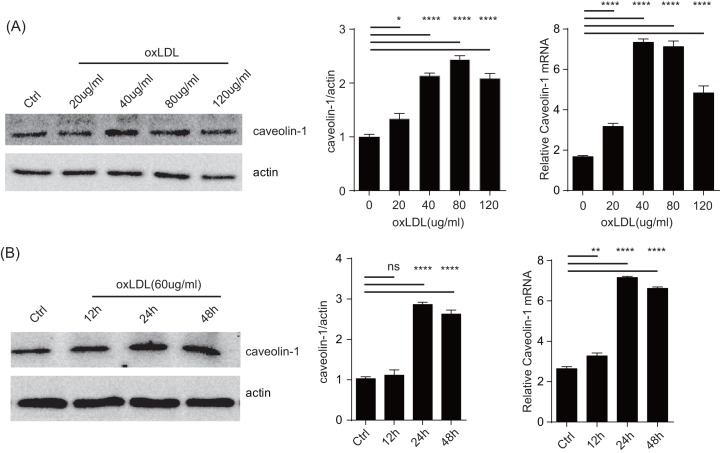
oxLDL promotes caveolin-1 production (**A**) Cultured RAW264.7 cells were incubated with indicated concentration of oxLDL for 24 h, caveolin-1 expression was analyzed by Western blots. Left: a representative blot (*n*=5). Middle: densitometry analysis of the blot. All readings are normalized to the ratio of caveolin-1 to actin at time zero (set as 1). Right: total RNA was analyzed by quantitative real-time PCR. The data are expressed as the fold change over the control value (*n*=5). *P<0.05 compared with the PBS-pretreated cells. (**B**) Similar to (A) RAW264.7 cells were treated with oxLDL (60 mg/ml) for different durations, caveolin-1 expression was measured as above (*n*=5). Left: protein levels; right: QPCR. *P<0.05 compared with the PBS-pretreated cells. *P<0.05, **P0.01, ***P0.001, ****P<0.0001

**Figure 2:**
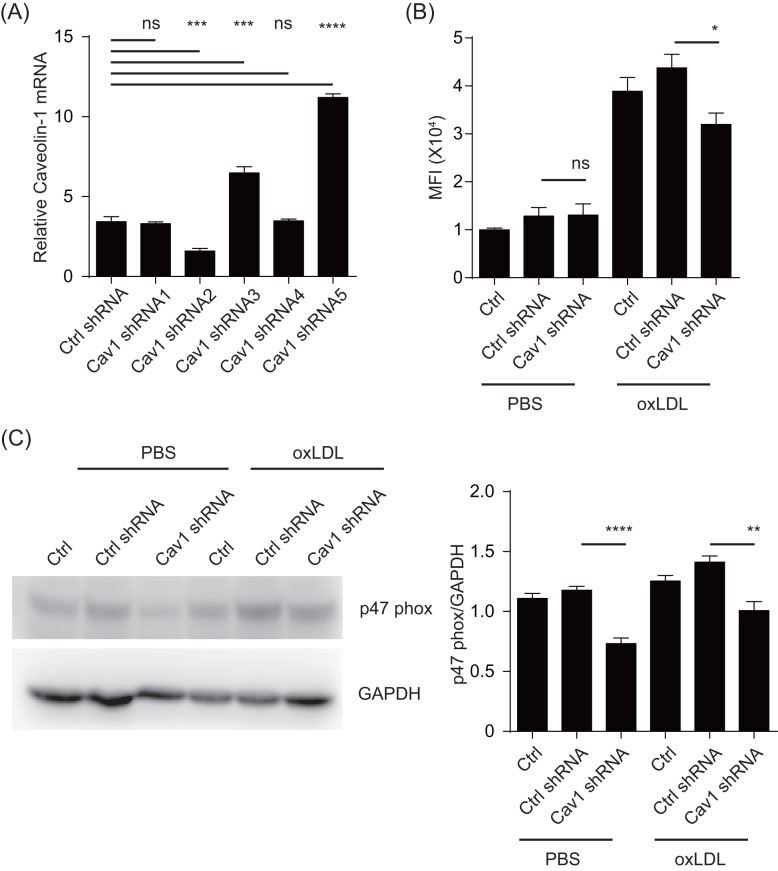
oxLDL promote ROS and p47phox with the caveolin-1 production (**A**) *Cav1* mRNA level in different shRNA lentiviral particle transfected group was measured by RT-PCR. Scramble shRNA transfection as negative control the data are expressed as a fold level of the control value and are the means ± S.D. for five separate experiments. *P<0.05 compared with the cells transfected with Ctrl shRNA. (**B**) Effects of caveolin-1 on oxLDL-stimulated ROS generation in Raw264.7 cells. ROS generation was measured as above (*n*=5). *P<0.05 compared with the cells transfected with Ctrl shRNA. (**C**) p47phox expression was measured by Western blots. The representative results of Western blots are shown. All readings are normalized to the ratio of caveolin-1 to actin at time zero (set as 1). **P<0.05 compared with the cells transfected with Ctrl shRNA. *P<0.05, **P0.01, ***P0.001, ****P<0.0001

In our previous analysis of eNOS regulation, we found that in addition to its well-characterized targetting to caveolin-1, the eNOS also blocked caveolin-1 production [17]. As eNOS is a key regulator of epithelial homeostasis, we wondered if the previous results implied that caveolin-1 level served as a balance control in oxidase responses. Using RAW264.7 as the model, we stimulated these cells with increasing amounts of oxLDL (Figure 1A,B). oxLDL treatment substantially increased the abundancy of caveolin-1 at the protein and mRNA levels in a dose–response manner from 20 to 80 μg/ml. Using a dose of 60 μg/ml, oxLDL stimulated the increase after 24 h, although the mRNA increase was seen after 12 h. Previously, it was reported that this increase was driven by ERK and MAPK expression [18], however how this up-regulation is related to downstream inflammatory consequences was not well understood. To that end, we produced a series of shRNA and tested their knockdown efficiency. Amongst them a particular construct reduced caveolin-1 expression levels (Figure 2A). This was accompanied by reduction in ROS (Figure 2B). The same phenomenon occurred in p47phox of both untreated and oxLDL-treated RAW264.7 cells (Figure 2C). These results suggest that caveolin-1 may signal to increase the protein levels of p47phox, creating a chain reaction to promote oxidative responses.

### oxLDL leads to cellular ageing

**Figure 3:**
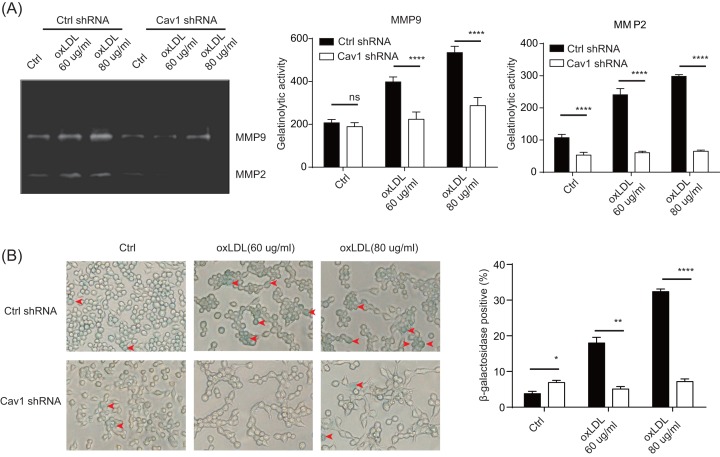
oxLDL leads to cellular ageing (**A**) RAW264.7 cells were treated with caveolin-1 shRNA or scramble shRNA as in Figure 1. The samples were stimulated with different doses of oxLDL as indicated. Twenty-four hours later, cell supernatants were collected for MMP2/9 activity determination. (**B**) RAW264.7 cells were transfected with caveolin-1 shRNA or scramble shRNA by lentivirus system. Then samples were stimulated with different doses of oxLDL as indicated. Twenty-four hours later, cell senescence was determined by β-gal staining. Upper panel, representative pictures of different treatments, red arrows indicate senescent cells; lower panel, statistical results (*n*=3). *P<0.05, **P0.01, ***P0.001, ****P<0.0001

Both oxLDL and caveolin-1 have been implicated in atherosclerotic and inflammation changes. To study whether oxLDL’s effect requires caveolin-1, we treated RAW264.7 cells with oxLDL and their matrix metalloproteinase MMP2 and MMP9 levels were studied [16,19]. At both 60 and 80 μg/ml, oxLDL promoted significant increase in MMP9 and MMP2, indicating a positive effect on inflammation (Figure 3A). While caveolin-1 shRNA did not completely block MMP9 production, the overall levels were proportionally reduced. MMP2 on the other hand, completely disappeared. In support of the involvement of caveolin-1 in senescence, β-gal production [20] upon oxLDL treatment was also significantly blocked by the caveolin-1 knockdown, confirming that the ability of oxLDL to induce cellular ageing requires caveolin-1 (Figure 3B).

### oxLDL promotes caveolin-1 and p47phox membrane colocalization

**Figure 4:**
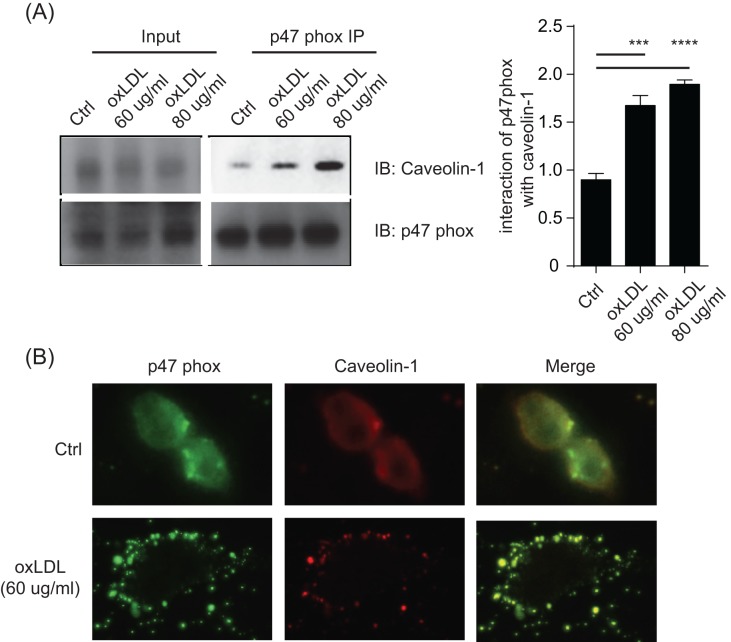
oxLDL promotes caveolin-1 and p47phox membrane colocalization (**A**) p47phox interacts with caveolin-1 in oxLDL induced cell senescence.RAW264.7 cells were stimulated with different doses of oxLDL as indicated. 4 h later, cells were lysed and pulled down by anti-p47 protein A/G agarose beads overnight. Samples were loaded for Western blotting analysis. The data prensented here are representative or means±S.D. of five independent experiments. (**B**) RAW264.7 cells were stimulated with 60 ug/ml oxLDL for 4 h. Cellular localizations of p47phox and caveolin-1 were analyzed by immunofluorescent imaging (400X). For p47 phox and caveolin-1 illumination, bandpass filter of 525/50nm and 700/75nm were used, respectively. The data prensented here are representative of five independent experiments. *P<0.05, **P0.01, ***P0.001, ****P<0.0001

NADPH oxidase is only functional in its membrane-associated forms [21,22]. In oxLDL-mediated activation, caveolin-1 is increased at the protein level (Figure 1). Currently, there is no knowledge whether caveolin-1 and p47phox can directly regulate each other. Should this be the case, it would suggest that p47phox is recruited to the membrane via caveolin-1, providing the necessary membrane targetting for its activation. Upon oxLDL stimulation, p47phox became associated with caveolin-1, as revealed by immunoprecipitation (Figure 4A). This result indicates caveolin-1 may serve as the ‘membrane address’ or a target for the membrane recruitment of p47phox. For visualization, we co-stained caveolin-1 and p47phox before and after oxLDL treatment. Before the treatment, both molecules appeared in diffused fashion. However, oxLDL led to a dramatic co-localization on the membrane (Figure 4B). Therefore, our results suggest that one of the effects of oxLDL is to regulate the association between caveolin-1 and p47phox, potentially initiating ROS production.

## Discussion

NADPH oxidases are a vital contributor to the ROS generation which plays an essential role in cellular senescence. As NOXs are functionally gated by the membrane recruitment, the regulatory events in this process are important study topics. On the other hand, genetic deletion of Caveolin-1 is associated with a significant increase in serum cholesterol levels and a significantly reduced rate of atherosclerosis [23]. A more precise model employing the overexpression of caveolin-1 in endothelium further confirmed its role in the disease [24]. These findings confirm the role of caveolin-1 in transcytosis of cholesterol into the subendothelial space. Yet, whether these two seemingly independent factors may function co-operatively is currently unknown. This work reveals a direct association between p47phox and caveolin-1, suggesting a new regulatory event in the pro-atherosclerotic activities of NOXs and caveolae.

As oxLDL requires caveolae for this internalization, the binding of oxLDL will likely to change the latter’s state of association with other signaling/structural moieties. In hepatocytes, TGF-β activates NOX1 in a Caveolin-1-dependent manner [25]. In that particular report, the association of src family kinases to the CSD is likely the molecular link leading to NOX1 activation. Whether such a similar process is at work for NOX2 is not known. Prior to this work, it was found by confocal imaging in THP-1 cells that p47phox showed increased membrane recruitment and caveolin-1 association following high glucose treatment [26]. A detailed fractionation study suggested that in the membrane highly enriched with caveolin-1 there is significant presence of actin cytoskeleton [27]. In another report, the actin cytoskeleton shows strong association with p40phox and p47phox subunits, and this association is a regulatory mechanism for neuronal NOX2 activities [28]. These observations may suggest a bridging mechanism for targetting p47phox to the membrane, i.e. via cortical cytoskeleton. On the other hand, as caveolae are a subset of the generically defined lipid rafts [21], and the lipid rafts have the natural tendency to gather PIP2 [29]. NOX may be attracted by PIP2 in the vicinity of caveolae. In a sophisticated analysis, a previous report demonstrated that p47phox is uniquely attracted via electrostatic interactions and used its PX domain to insert hydrophobic residues into the membrane [30]. In spite of these indirect associations, in this work we were able to show a direct association of these two proteins, as analyzed by co-immunoprecipitation, suggesting a direct molecular association and further confirming the results by Hayashi et al. [26]. Further understanding the molecular details of this association may require detailed mutational mapping of both molecules. Nevertheless, previous reports and our current analysis suggest several possible routes whereby p47phox is targetted to the membrane, which serves as a promising target for therapeutic intervention.

## Abbreviations

β-gal: β-galactosidase
CSD: caveolin scaffolding domain
GAPDH: glyceraldehyde phosphate dehydrogenase
MAPK: mitogen-activated protein kinase
oxLD: oxidative low density lipoprotein
ROS: reactive oxygen species
